# Genetic diversity and population structure of parasite infrapopulations within and across hosts for two trophically transmitted trematode parasites

**DOI:** 10.7717/peerj.19178

**Published:** 2025-04-28

**Authors:** Sarah R. Goodnight, April M.H. Blakeslee, Michael W. McCoy

**Affiliations:** 1 Department of Environmental Science and Policy, George Mason University, Woodbridge, VA, United States of America; 2 Department of Biology, East Carolina University, Greenville, NC, United States of America; 3 Department of Biology, Florida Atlantic University (Harbor Branch Campus), Fort Pierce, FL, United States of America

**Keywords:** Genetic diversity, Trophic level, Parasite, Freshwater pond, Genetic population structure, Digenean, Infrapopulation

## Abstract

Complex parasite life cycles frequently require trophic transfer of parasites from an intermediate host prey to a definitive host predator. This results in aggregated distributions of parasites in predator host populations, which are subsequently expected to host more genetically diverse parasite infrapopulations than lower trophic level hosts. Host dispersal and seasonal population dynamics, particularly in the case of first-intermediate hosts, are also expected to drive population genetic patterns within and across populations. To examine how parasite life history and host ecology influence parasite genetic patterns, we characterized the genetic diversity of within-host infrapopulations, as well as overall population genetic structure, of sympatric tongueworm (*Halipegus occidualis*) and lungworm (*Haematoloechus complexus*) freshwater trematode parasite populations. Parasites were collected across three host stages (snail, odonate insect, and frog) and sequenced at the cytochrome oxidase I (COI) mitochondrial region (519 bp for lungworms; 526 bp for tongueworms) to characterize genetic variation within and across hosts. Infection abundance per host and genetic diversity of within-host parasite infrapopulations generally increased with host trophic level, as expected. Additionally, tongueworm assemblages in odonate hosts were essentially equally as genetically diverse (depending on the index used) as those in definitive host frogs; tongueworms have an additional trophic transfer in their life cycle before the odonate stage, which highlights how trophic transmission and multi-host life cycle structure can benefit parasites by increasing genetic diversity of sexually reproducing adult assemblages. We also found that tongueworm populations, which infect a long-lived snail as a first-intermediate host, had higher population genetic diversity than lungworms, which infect a much shorter-lived snail with highly unstable population dynamics. Thus, we expect that first-intermediate host dynamics and dispersal ability played a large role in predicting population-level parasite genetic diversity and genetic structure in this system. This study investigates the effects of small- and large-scale processes on parasite genetic population structure and diversity and provides critical genetic data for future studies on these genera.

## Introduction

Parasites and pathogens are critical components of food webs around the globe; in some systems, parasitic species make up more biomass than free-living species (Kuris et al., 2008; Lambden & Johnson, 2013). Consequently, parasites are increasingly recognized as important indicators of, and active contributors to, ecosystem health (Lafferty, 1997; Hudson, Dobson & Lafferty, 2006). Perhaps the most dynamic examples of the pervasive effects of parasites on food webs are parasites that evolved multi-host (*i.e.,* complex) life cycles. These parasites must pass sequentially through multiple host species to complete their life cycles, which often include distinct asexual and sexual reproductive stages in different host taxa (Gibson & Bray, 1994).

Digenean trematodes, or flukes, are ecologically and economically important metazoan macroparasites that exhibit this type of complex life history strategy (Gibson, 1987; Gibson & Bray, 1994; Niewiadomska & Pojmanska, 2011). Transitions between hosts often correspond with transitions between life stages that can only be accomplished after a trophic transfer from infected prey to a competent host predator (Kuris, 1997; Lafferty, 1999; Poulin, 2010). Trophically transmitted parasites accumulate within predator hosts over time as more infected prey are consumed (Crofton, 1971; Karvonen et al., 2004; Lester & McVinish, 2016). Thus, the digenean life history strategy often leads to aggregated parasite distributions, where a few host individuals carry a large portion of the parasite population (Poulin, 2013; Lester & McVinish, 2016). Digenean trematodes can also accumulate up the food chain as they are passed from prey to predator, leading to greater parasite abundances in higher trophic level hosts relative to hosts at lower trophic levels (Chen et al., 2008; Lester & McVinish, 2016). In such systems, second-intermediate hosts that are prey to definitive hosts serve as a link between the parasite’s asexual and sexual life stages. Specifically, second-intermediate hosts collect encysted parasites from multiple first-intermediate hosts and then transfer cohorts of potentially genetically-distinct parasites to the final host—where parasite sexual maturation and reproduction occurs (Choisy et al., 2003; Keeney, Waters & Poulin, 2007a). Most adult digenean trematodes are hermaphroditic and can even self-fertilize (Niewiadomska & Pojmanska, 2011), but sexual reproduction with unrelated partners is a key mechanism for enhancing genetic diversity (Brown et al., 2001; Rauch, Kalbe & Reusch, 2005). Therefore, the aggregation of parasites in individual hosts *via* trophic transfer not only ensures the opportunity for eventual sexual reproduction in the final host, but directly facilitates genetic diversity in the next generation (Brown et al., 2001).

Over larger spatial scales, the genetic structure and diversity of parasite populations is highly influenced by host dispersal (Blasco-Costa, Waters & Poulin, 2012; Blasco-Costa & Poulin, 2013; Mazé-Guilmo et al., 2016; Penczykowski, Laine & Koskella, 2016; Matthee, 2020). Parasites with complex life cycles have multiple pathways of dispersal *via* first-intermediate, second-intermediate, or definitive hosts. Different types of hosts colonize new habitat patches carrying different life stages of the parasite, which can establish new parasite populations given that all other obligate hosts are present post-colonization (Thrall & Burdon, 1997; Penczykowski, Laine & Koskella, 2016). For parasites infecting hosts that tend to occupy discrete habitat patches, such as ponds and lakes, gene flow between subpopulations is critical for maintaining genetic diversity both within and across habitat patches (Blouin et al., 1995; Sire et al., 2001b; Flores et al., 2013). First-intermediate hosts can typically carry only one or a few genotypes of parasite and are more dispersal-limited than more mobile, higher trophic level hosts. Thus, when parasites are introduced into new populations *via* dispersal of an infected first-intermediate host, the genetic diversity of the parasite population will be limited (*i.e.,* founder effects) (Barton & Charlesworth, 1984; Blakeslee, Byers & Lesser, 2008; Blakeslee et al., 2020). In contrast, when parasites are dispersed to new habitat patches *via* mobile definitive hosts, founder effects may be less prominent because individual definitive hosts are often infected with multiple genetically-distinct parasites (Rauch, Kalbe & Reusch, 2005; Blasco-Costa, Waters & Poulin, 2012; Blakeslee et al., 2020). Indeed, the genetic diversity of parasite populations is typically driven by migration rates of the definitive hosts among habitat patches (Ortego et al., 2008; Mazé-Guilmo et al., 2016; Penczykowski, Laine & Koskella, 2016). Therefore, host mobility and dispersal rates are expected to interact with host trophic level and parasite aggregation to influence the genetic structure of parasite populations.

The aim of this study is to investigate how patterns of genetic diversity in two ecologically similar parasite species that inhabit patchy habitats (freshwater ponds) are differentially influenced by host and parasite life histories. We tested the prediction that host trophic level would predict within-host parasite genetic diversity, specifically that higher trophic level hosts would contain more genetically diverse parasite assemblages due to the accumulation of genotypes in successive hosts through obligate trophic transfers. In contrast, at the population scale, we tested the prediction that the relative isolation of the population and the dispersal ability of the hosts would drive overall genetic diversity. Specifically, we expected that populations of parasites that require rare, dispersal-limited hosts would have lower population diversity than parasites that have common hosts that frequently disperse among habitat patches.

### Study system

**Figure 1:**
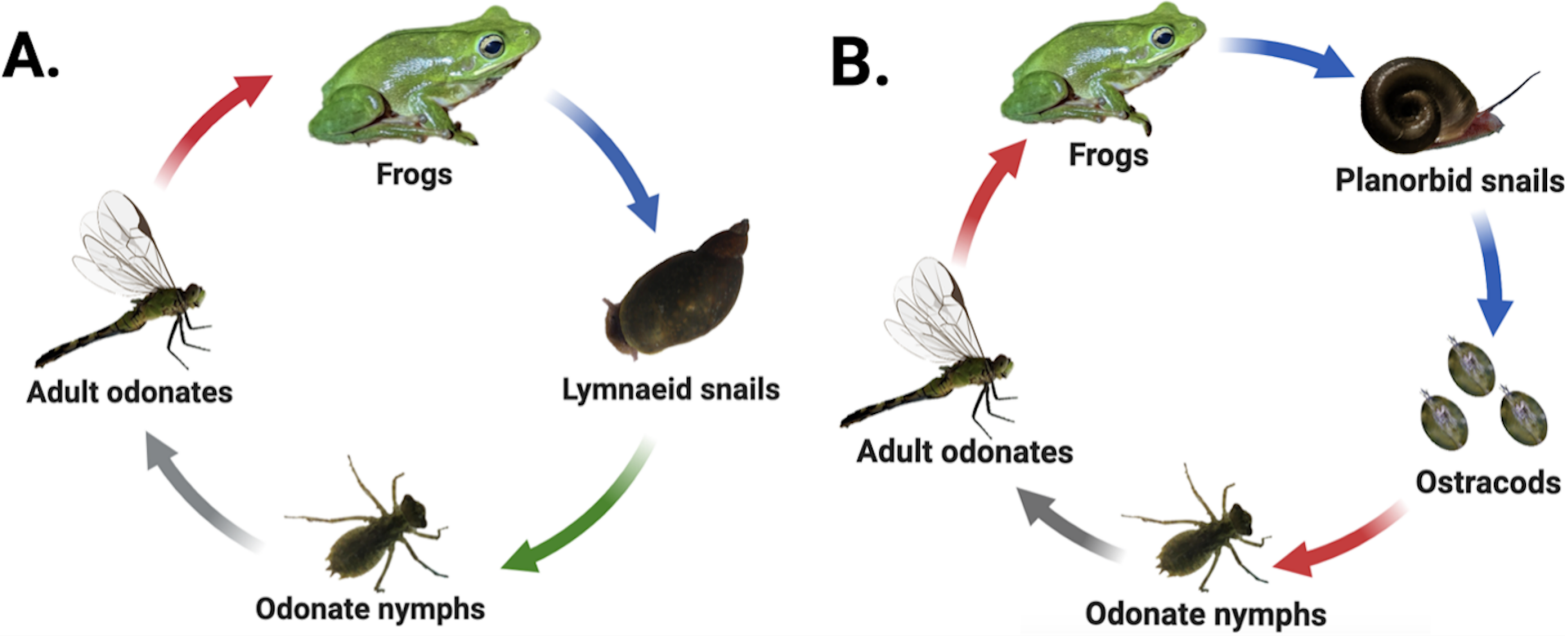
Life cycles of focal parasites. Arrow colors correspond to life stage transitions, *i.e.,* red, trophic transmission; blue, passive transmission; green, active transmission; and gray, host movement to terrestrial environment *via* emergence. *Haematoloechus complexus* lungworms (A) are common and have a contiguous range with frequent colonization of new habitat patches, while *Halipegus occidualis* tongueworms (B) are rarer parasites with highly spatially isolated populations on the U.S. east coast. Key life history differences include first-intermediate host species (lymnaeid or physid snails *vs.* planorbid snails), number of trophic transfers (two *vs.* one), number of hosts (four *vs.* three), and passive (tongueworms) *vs.* active (lungworms) cercarial stages. Figure reproduced in part from Goodnight & McCoy (2024) with permission. Diagrams created in Biorender (biorender.com).

Many digenean trematodes have evolved to infect host species that inhabit patchy freshwater pond habitats characterized by fluctuating hydroperiods and variable seasonal dynamics. Consequently, movement of parasites among habitat patches commonly depends on the movements of often dispersal-limited hosts (Vizoso, Lass & Ebert, 2005; Johnson et al., 2016; Moss et al., 2020). Our study examined two freshwater digenean trematode species found in eastern North Carolina that are also broadly distributed throughout the United States. The first, *Haematoloechus complexus* (hereafter “frog lungworm”), has a three-host life cycle that passes through both aquatic and terrestrial hosts (Krull, 1933) (Fig. 1A). Lymnaeid (generally *Pseudosuccinea* sp.) or sometimes physid (*Physa* or *Physella* sp.) snails are obligate first-intermediate hosts within which the parasite develops into sporocysts and undergoes asexual replication, releasing active, free-swimming larval cercariae into the water column which seek out aquatic odonates (dragonfly and damselfly nymphs) and other aquatic insects as second-intermediate hosts. Cercariae encyst within the second-intermediate hosts’ tissues as metacercariae (Snyder & Janovy Jr, 1994) and undergo several weeks of development as cysts. The insect hosts ultimately emerge from the pond as adults, where they are consumed by frogs, the parasite’s final (*i.e.,* definitive) host. The parasites excyst in the frog’s stomach and mature into adults within the frog’s lungs before sexually reproducing (Krull, 1933; Snyder & Janovy Jr, 1994).

The second parasite, *Halipegus occidualis* (hereafter “frog tongueworm”), has a unique four-host life cycle with a similar suite of intermediate and definitive hosts, but also with the inclusion of a paratenic (= transport) host (Fig. 1B) (Zelmer & Esch, 1998a). Planorbid snails, generally *Planorbella* (formerly *Helisoma*) sp., are the obligate first-intermediate host within which tongueworm sporocysts and rediae develop and produce cercariae, while zooplankton (primarily Ostracoda) are the second-intermediate host. Zooplankton encounter passive, immobile cercariae released from infected snails and consume them while foraging in the water column. Once ingested, the cercariae encyst in the zooplankton’s tissues as metacercariae (Zelmer & Esch, 1998b). Odonate nymphs consume infected zooplankton, and for this trematode species they function as a paratenic host in which no obligate parasite development takes place (Zelmer & Esch, 1998a). Instead, the odonate nymphs collect metacercariae in their gastrointestinal tracts before emerging from the pond and being consumed by frogs (Thomas, 1939; Zelmer & Esch, 1998a; Bolek, Tracy & Janovy Jr, 2010). In most host frog species (except the American bullfrog where they remain in the gut, (Stigge & Bolek, 2016), the parasites excyst and develop in the stomach before moving to the frog’s buccal cavity where they cluster under the tongue or in the throat and sexually reproduce (Zelmer, Wetzel & Esch, 1999).

These two parasite species are an ideal study system to examine the impacts of host life history characteristics and trophic transmission on parasite genetic diversity because they share several host species and are often found in sympatry in the eastern USA (Wetzel & Esch, 1996); yet they have several key differences in the structure of their life cycles, host dispersal ability, and spatial distribution. Specifically, the first-intermediate host of lungworms, *Pseudosuccinea* and *Physa* (or *Physella*) sp. snails, are very common, have high rates of dispersal, and are highly successful at establishing new populations when colonizing new habitat patches—indeed, both snail taxa are considered invasive species on several continents (De Kock, Joubert & Pretorius, 1989; Pointier et al., 2007; Albrecht et al., 2009; Vinarski, 2017; Ebbs, Loker & Brant, 2018; Ngcamphalala, Malatji & Mukaratirwa, 2022). In contrast, the first-intermediate host of tongueworms, ramshorn snails (*Planorbella* sp.), are a larger and less common snail in the eastern U.S. and have lower dispersal and colonization rates than other aquatic snails (Fernandez et al., 2010; Martin et al., 2020). Ramshorn snails can be uncommon in the southeastern U.S., where the mild acidity of many ponds makes them unsuitable for moderately large aquatic snail species with specific mineral requirements (Sutter & Kral, 1994; Thorp & Covich, 2001). Thus, frog tongueworms are more likely to occur in highly isolated populations in eastern North Carolina while frog lungworms are widespread, with presumably high gene flow between ponds, making this system optimal for a comparative study on the impacts of host abundance, life history, and dispersal ability on parasite genetic diversity.

For this study, we used a fragment of the barcoding gene cytochrome oxidase I (COI) from parasites collected from snail, odonate, and frog host stages to elucidate patterns of genetic diversity across hosts in natural tongueworm and lungworm populations. We directly measured within-host parasite genetic diversity (*i.e.,* diversity of parasite infrapopulations contained within the same host individual) for both parasite species to investigate how parasite diversity changes as host trophic level increases. We also measured overall population-level genetic diversity, structure, and neutrality metrics of the different parasite populations to investigate how spatial isolation and assumed gene flow from differential host dispersal informs parasite population genetic structure, history, and population divergence. We predicted that for both lungworm and tongueworm parasite populations, hosts at higher trophic levels would harbor parasite infrapopulations with higher genetic diversity than hosts at lower trophic levels. We also predicted that tongueworm populations would be overall less genetically diverse than lungworm populations, due to the expected relative isolation of tongueworm populations as compared to the more common and cosmopolitan lungworm species.

Despite their importance to freshwater ecosystems, comprehensive genetic studies of trematode parasites that include samples from hosts across an entire life cycle are uncommon, especially for historically overlooked taxa like *Haematoloechus* spp. lungworms and *Halipegus* spp. tongueworms. Studies characterizing the genetic structure of parasite infrapopulations contained within single host individuals are particularly rare, yet are critical for fully understanding the genetic population structure of macroparasites (Sire et al., 2001a; Vilas, Paniagua & Sanmartín, 2003).

## Materials & Methods

### Sample collection

Hosts and parasites were collected from two freshwater ponds <5 miles apart in eastern North Carolina during the summer months (May–August) over the course of two years (2021–2022). Both focal parasite species and their respective snail hosts co-occurred at one site (Lowe Pond), while only *H. complexus* lungworms and its host snails *Pseudosuccinea* sp. and *Physa* sp. were found at the second site (Bell Pond). We did not collect or process any zooplankton (ostracod) hosts from either pond due to logistical constraints, since dissecting enough individual zooplankton to characterize parasite population structure was not feasible within the scope of this work.

Odonate nymphs and snails were collected using a dipnet, and emerging dragonfly and damselfly adults were collected by hand from emergent vegetation during evening surveys at each site. All collected odonates were frozen whole at −20 °C, while all *Pseudosuccinea* sp. and *Physa* sp. snails were maintained alive in freshwater aquaria before dissection. When sampling *Planorbella* sp. ramshorn snails, the first-intermediate host for tongueworms, we prioritized non-destructive sampling measures since (a) *Planorbella* as well as the tongueworm parasite itself are rare taxa in North Carolina, and may be vulnerable to steep population declines (SR Goodnight, 2021, unpublished data; Russell et al., 2015), and (b) the population density of the ramshorn snails was observed to be very low (Table 1). Thus, we dissected only a random subset (*n* = 9) of collected ramshorn snails. To identify infections in the remaining ramshorns (*n* = 10), and to collect tongueworm parasite material for genetic analysis, captured snails were brought to the lab and placed in individual 50 mL Falcon tubes with spring water for 24 h, after which we examined an aliquot for shed cercariae (identified according to Krull, 1935 and Goater, Browne & Esch, 1990). If snails were deemed infected using this cercarial shedding method, the snails were removed from the Falcon tubes which were then spun in a centrifuge to aggregate all shed cercariae into a pellet. The pellet was stored at −20 °C until DNA extraction. Snails were assumed to be uninfected if no cercariae were observed. The accuracy of this method for identifying trematode infections as compared to dissection is highly dependent on trematode species (Curtis & Hubbard, 1990; Born-Torrijos et al., 2014), but although we may have missed a small proportion of infections, we still identified a relatively high proportion of infections in the collected snails (Table 1). These high infection rates were consistent with previous studies on pond populations of this parasite (Crews & Esch, 1986). Infected snails were maintained in the lab environment for 2–3 days, refreshing the water and collecting cercariae each day, after which snails were re-released at the original site of capture.

**Table 1:** Parasite infection prevalence and mean infection abundances per host for all life stages. *Physa* sp. snails (*n* = 740) are not included in this table or in any analyses since we did not find *H. complexus* lungworms in physids from either field site. Tongueworm prevalence and infection abundance were calculated using hosts from site LOWE only, while lungworm prevalence and infection abundance were calculated for both sites.

**Host taxa**	**N**_**total**_		**Lungworm** **N**_**infected**_		**Tongueworm** **N**_**infected**_	**Tongueworm prevalence ± SD**	**Lungworm prevalence ± SD**		**Tongueworm infection abundance** **(mean ± SD)**	**Lungworm infection abundance** **(mean ± SD)**	
	**BELL**	**LOWE**	**BELL**	**LOWE**	**LOWE**	**LOWE**	**BELL**	**LOWE**	**LOWE**	**BELL**	**LOWE**
***Pseudosuccinea*** **snails**	283	171	19	57	–	–	0.07 ± 0.26	0.33 ± 0.48	–	–	–
***Planorbella*** ** snails**	0	19	–	–	8	0.42 ± 0.49	–	–	–	–	–
**Odonates**	238	291	46	76	32	0.11 ± 0.24	0.19 ± 0.40	0.26 ± 0.44	0.13 ± 0.62	0.47 ± 1.54	8.16 ± 53.92
**Frogs**	26	26	21	13	15	0.58 ± 0.46	0.81 ± 0.40	0.50 ± 0.50	1.02 ± 2.20	8.54 ± 12.03	2.01 ± 4.57

Adult frogs at both sites were captured by hand during active breeding nights, and species, mass, and sex were recorded for each individual upon capture. We captured primarily American green tree frogs (*Dryophytes cinereus*), since they were the most abundant species at both ponds, but also collected individuals from two species of ranid frog: southern leopard frogs (*Lithobates sphenocephalus*) and green frogs (*Lithobates clamitans*). Upon capture, buccal cavities of all frogs, regardless of site or species, were examined for tongueworms by gently opening the mouth with a flat spatula, after which any visible flukes were removed with forceps and transported to the laboratory alive before being frozen at −20 °C for genetic analysis. A subset of frogs (*n* = 18) were then re-released upon examination of the buccal cavity and removal of tongueworms, to reduce impact on the frog population, while the remainder (*n* = 52) were humanely euthanized at the field site (Edwards, Steele & Gordon, 2017) and stored whole at −20 °C until dissection to quantify lungworm abundance and immature tongueworms retained in the stomach. Collection of frogs and parasites was covered under ECU IACUC Animal Use Protocol #D357, FAU IACUC protocol #A22-16, and North Carolina Wildlife Collection Permit #22-SC01489.

### Specimen dissection and morphological identification

*Pseudosuccinea* and *Physa* spp. snails were dissected by first crushing each snail with flat forceps, then examining the internal tissues under a dissecting scope for visible sporocysts and mature cercariae. Upon discovery of an infection, parasite tissues were separated from host tissue using a micropipette and frozen whole at −20 °C until further processing. Lungworm infections were identified based on cercarial morphology and snail host species according to Krull (1933). *Planorbella* ramshorn snail dissections were accomplished in the same manner and identified according to Krull (1935).

Odonate nymphs and adults were identified to species when possible, or to genus for early instars, upon dissection. Since tongueworm metacercariae exclusively inhabit the gut of odonates, and lungworm metacercariae may be found anywhere in the body (Wetzel & Esch, 1996), odonates were dissected by first removing the gastrointestinal tract and associated viscera from each individual using a scalpel and curved forceps, and then examining the contents under a dissecting scope. After thorough examination of the viscera, the remaining tissue for each odonate was flattened between two microscope slides which were then clamped to allow easy visualization of any metacercariae in the respiratory tissues, exoskeleton, or limbs. Lungworm and tongueworm metacercariae were identified based on morphology and placement in the body of the nymph according to Krull (1933) and Snyder & Janovy Jr (1994) for lungworms, and Krull (1935) and Wetzel & Esch (1996) for tongueworms. All metacercariae were separated from host tissues using thin forceps and a micropipette and individually frozen at −20 °C.

Frogs were dissected by first removing the internal organs from the body cavity and excising the lungs with a scalpel, which were then compressed between two microscope slides to visualize lungworms. Stomach and intestines were placed in a Petri dish, and contents were flushed out with distilled water and examined under a dissecting scope to locate any immature flukes still residing in the gut, since both focal parasite species mature in the stomach before moving to the lungs or mouth (Krull, 1933; Shields, 1987; Stigge & Bolek, 2016). We additionally examined the buccal cavity and throats of all frogs again to ensure all mature tongueworms had been successfully removed during the field collections. All flukes regardless of maturity were separated from host tissue or gut contents with forceps and refrozen at −20 °C for genetic analysis. Lungworm flukes were identified according to Kennedy (1981), while tongueworms were identified according to Krull (1935) and Stigge & Bolek (2016).

### Molecular analysis

At the first-intermediate host stage, we pooled trematode tissue (cercariae, sporocysts, and/or rediae) from each individual snail, resulting in one sequence per snail individual. We took this approach for several reasons. For one, though first-intermediate host snails could conceivably host multiple genotypes of trematode if simultaneously infected by multiple miracidia (Rauch, Kalbe & Reusch, 2005; Keeney, Waters & Poulin, 2007a), this variation would not commonly be detected using the cytochrome oxidase subunit I (COI) mitochondrial marker we employed in this study (see Correia et al., 2023). Second, since trematodes go through larval duplication (asexual reproduction) in their snail hosts, it is most typical at this stage of the life cycle to detect highly clonal assemblages of parasites with very low genetic diversity (Rohde, 2005; Keeney et al., 2008a; Keeney et al., 2008b; Keeney et al., 2022). Third, to characterize genetic variation within individual snail hosts, it would have been necessary to reliably obtain sequence data from many different individual sporocysts/rediae/cercariae per snail; this proved unfeasible for this work and would have been unlikely to reveal significantly different diversity patterns than our pooling approach. In contrast, for second-intermediate and definitive host stages, where trematodes may accrue from multiple divergent host individuals and geographically separated host populations, genetic variation is much more likely in individual metacercariae and adults. Thus, for insect and frog samples, we separated and processed individual metacercariae and adult worms in order to capture multiple parasite sequences per host individual.

We extracted and concentrated DNA from trematode samples using a standardized cetyl trimethyl ammonium bromide (CTAB) alcohol precipitation method (following France, Rosel & Ewann, 1996; Blakeslee, Byers & Lesser, 2008 protocols). A partial region of the cytochrome oxidase subunit I (COI) mitochondrial gene was amplified using the universal trematode primer set JB3 (5′-TTTTTTGGGCATCCTGAGGTTTAT-3′) and COI R-Trema (5′-CAACAAATCATGATGCA AAAGG-3′) (Miura et al., 2005). This gene and primer set have been previously established as effective for delineating haplotype diversity in other trematode populations (Miura et al., 2005; Urabe & Shimazu, 2010; Urabe, Nishimura & Shimazu, 2012). We used two separate PCR profiles: a “standard” profile for large adult worms and sporocyst/rediae tissue from snails, and an “extended” profile for small or immature adult worms and individual metacercariae, which had additional cycles to account for the small amount of biomass in those samples (Kramer & Coen, 2001). The standard profile was: 95 °C for 2 min; 30 cycles of 95 °C for 30 s, 55 °C for 30 s, and 72 °C for 60 s; and 72 °C for 5 min (Steinberg, Krimsky & Epifanio, 2008). The extended profile was modified from Steinberg, Krimsky & Epifanio (2008) to consist of: 95 °C for 2 min; 40–45 cycles of 95 °C for 30 s, 55 °C for 30 s, and 72 °C for 60 s; and 72 °C for 5 min. Successful PCR product was sent for purification and Sanger sequencing to Psomagen, USA (Rockville, MD, USA).

Sequences were aligned and inspected for discrepancies using Geneious Prime 2023.0.1 (Biomatters Ltd). Alignments were accomplished using the ClustalW algorithm and aligned without gaps (Larkin et al., 2007; Sievers et al., 2011). We obtained COI sequences of 519 bp for lungworms (*n* = 126 sequences) and 526 bp for tongueworms (*n* = 195) and searched the reference sequences of both species’ alignments using BLAST (Benson et al., 2009) to find similarities to published sequences in GenBank and confirm species identification. Our lungworm sequences matched with 100% identity to a 349 bp *Haematoloechus complexus* sequence from León-Règagnon (2010) (accession no. HQ141702). Our tongueworm sequences did not match with anything above 85% identity on GenBank, since this species has not been sequenced at this gene region previously, but our closest match was a 507 bp sequence of *Allogenarchopsis problematica* (a species also in the family Derogenidae) at 83.6% identical (accession no. AB828006; Urabe & Shimazu, 2010). Sequences were deposited in Genbank (accession no. PQ675873 –PQ675998 for lungworms and no. PQ668820 –PQ669020 for tongueworms).

### Sequence analysis and genetic diversity

For all genetic analyses, lungworm sequences from both Bell Pond and Lowe Pond were pooled together and analyzed as a group due to low sample sizes from certain life stages making inference difficult (see Table 2). Since genetic divergence between the two ponds was negligible (non-significant population *F*_ST_ = −0.00179, Table S1), we are confident that there is high gene flow between the two ponds, or that they were each relatively recently colonized from the same founding population, and thus can be considered one population. Haplotype networks were inferred using TCS v1.21 and plotted for visualization in PopArt (Leigh & Bryant, 2015). Haplotype rarefaction curves and estimated haplotype richness (*h*_*est*_) were calculated using EstimateS (Version 9; R. K. Colwell, http://purl.oclc.org/estimates; Colwell, Coddington & Hawksworth, 1997) and plotted using the R package ggplot2 (Wickham, 2016). Diversity metrics at both the host-level and the population-level were calculated using Arlequin v3.5 (Excoffier & Lischer, 2010) and the ape, pegas, vegan, and spider packages in R (Paradis, 2010; Brown et al., 2012; Oksanen et al., 2013; Paradis & Schliep, 2019). Specifically, we first calculated “within-host” diversity metrics using sequences from multiple parasites collected from the same host, essentially treating each host as its own infrapopulation of parasites. We then calculated separate “population-level” diversity metrics to characterize the large-scale genetic structure of each parasite population at each pond inclusive of all hosts and all life stages. For both approaches, haplotype diversity (*h*), haplotype richness (*h*_*rich*_), and Shannon–Weiner diversity (H’) were calculated using R while nucleotide diversity (*π*), gene diversity, pairwise differences, *F*_ST_ values, and theta values (Theta H, Theta pi, and Theta S) were calculated using Arlequin. We also calculated Tajima’s D and Fu’s Fs neutrality values with corresponding statistical summaries for both parasite populations using Arlequin (Excoffier & Lischer, 2010).

**Table 2:** Population- and host-level genetic diversity indices for sampled tongueworm (*Halipegus occidualis*) and lungworm (*Haematoloechus complexus*) parasite populations. Per parasite species and life stage, N_host_, total number of hosts sampled for genetic analyses; N_par_, total number of parasite sequences obtained; *h*_rich_, mean parasite haplotype richness per host; *h*_est_, estimated haplotype richness from rarefaction analysis per life stage; *h*, mean parasite haplotype diversity per host; and *π*, mean nucleotide diversity per host. Estimated haplotype richness was calculated using all hosts per life stage rather than per host individuals. For both population-level analyses (denoted by asterisks), all metrics were calculated inclusive of all life stages and all hosts, for the parasite population as a whole (*i.e.*, *h*_rich_, total population haplotype richness; *h*, total population haplotype diversity; and *π*, total population nucleotide diversity). Neutrality values and associated pvalues are also reported for both overall populations; significant values are bolded.

**LIFE STAGE**	**N**_**host**_	N_**par**_	***h***_**rich**_ ± SD	*h*_*est*_ ± SD	*h* ** ± SD**	*π* **± SD**	**Tajima’s** ***D***	***p***_**D**_	**Fu’s** ***Fs***	***p***_**Fs**_
***Tongueworms***										
Snails (sporocysts & cercariae)	11	11	1.0 ± 0.0	–	0.0 ± 0.0	0.00 ± 0.00				
Odonates (metacercariae)	12	19	1.42 ± 0.79	11.13 ± 6.41	0.21 ± 0.38	0.002 ± 0.004				
Frogs (adult worms)	24	165	2.67 ± 1.31	25.74 ± 12.40	0.46 ± 0.38	0.005 ± 0.004				
All tongueworm life stages*	47	195	18	57.21 ± 44.82	0.68 ± 0.031	0.008 ± 0.004	1.12	0.88	0.795	0.44
***Lungworms***										
Snails (sporocysts & cercariae)	26	26	1.0 ± 0.0	–	0.0 ± 0.0	0.00 ± 0.00				
Odonates (metacercariae)	23	75	1.43 ± 0.79	26.22 ± 22.05	0.216 ± 0.339	0.004 ± 0.018				
Frogs (adult worms)	10	25	1.70 ± 0.95	5.90 ± 1.69	0.366 ± 0.429	0.0015 ± 0.002				
All lungworm life stages*	58	126	11	20.86 ± 9.96	0.362 ± 0.054	0.003 ± 0.002	**−2.65**	** 0.00**	**−4.26**	** 0.051**

### Phylogenetic analysis

Phylogenetic reconstructions of the tongueworm and lungworm populations using COI sequences were constructed using Mr. Bayes (Ronquist & Huelsenbeck, 2003) implemented in Geneious version 2023.0.1 (Kearse et al., 2012). All sequences from both species were collapsed into unique haplotypes before each analysis. The substitution model was set to GTR with four gamma categories with default MCMC parameters, and the priors were set to a molecular clock with uniform branch lengths with a gamma of (1,1) and a shape parameter exponential of 10 (Ronquist & Huelsenbeck, 2003). For tongueworms, we obtained a 526 bp sequence from a sporocyst sample of its congener *Halipegus eccentricus*, which we collected from snails from a nearby pond, to use as the outgroup to root the tree; for lungworms, we used a 336 bp sequence from its congener *Haematoloechus varioplexus* taken from GenBank (accession no. MG647799, Leon-Regagnon & Topan, 2018) as the outgroup. Additionally, for tongueworms only, we constructed and tested a series of separate Bayesian trees using the same methodology as above using a subset of samples (*n* = 158) from which we were able to obtain longer reliable sequence lengths (905 bp). We did this extra analysis opportunistically for the tongueworm population to increase the amount of potential phylogenetically informative sites, thereby increasing confidence in the topology of the population tree.

### Statistical analysis

All model-based statistical analyses were performed in the R statistical programming environment (R Core Team, 2013). For all generalized linear models (GLMs) and generalized linear mixed models (GLMMs), we used the R packages glmmTMB (Magnusson et al., 2017) and lme4 (Bates et al., 2009) to fit initial models, and the function ICtab in package bbmle (Bolker, 2017) to perform model comparisons based on AIC scores. Models were interrogated and model fit was evaluated using the packages DHARMa (Hartig & Hartig, 2017) and sjPlot (Lüdecke, 2018a). The packages ggeffects (Lüdecke, 2018b) and emmeans (Lenth et al., 2019) were used to generate model predictions, and ggplot2 (Wickham, 2011; Wickham, 2016) was used for graphical summaries of model results.

We first investigated whether host taxa predicted tongueworm parasite infection abundances per host, using host type (odonate or frog) as our response variable. We did not include data from snail hosts in this analysis since we simply quantified snail infections as present or absent in all cases. Importantly, we only included parasite abundance data from frogs that were euthanized and dissected in this analysis, not the subset that were re-released (*n* = 18), since we were not able to quantify the entirety of the parasite community in released hosts; tongueworms collected from the buccal cavities of released hosts were only used for genetic analyses. To account for overdispersion in parasite counts, we compared fits of Poisson, negative binomial, and zero-inflated negative binomial GLMMs. We included only data from Lowe Pond for the analysis of tongueworm infection abundance since we did not find any tongueworms nor their obligate first-intermediate *Planorbella* snail host at Bell Pond. We repeated the same analysis separately for the lungworm populations, this time using data from both Bell and Lowe ponds since lungworms were found at both sites. For this analysis we tested pond site as an additional fixed effect alongside host taxa to account for differences in lungworm infection patterns between ponds.

We then investigated how host trophic level predicted within-host parasite genetic diversity for both parasite species by fitting four sets of GLMs with host taxa as a fixed effect and four different metrics of parasite genetic diversity as response variables: nucleotide diversity (*π*), haplotype richness (*h*_*rich*_), haplotype diversity (*h*), and Shannon–Weiner diversity (H’). All diversity metrics were calculated per individual host for this approach. Importantly, we again excluded snails from all analyses of diversity metrics and only tested odonates *vs.* frogs because we only obtained one sequence per snail host (see Methods); additionally, digenetic trematodes reproduce asexually in the snail host and thus generally show little to no genetic diversity at the haplotype level (Keeney, Waters & Poulin, 2007a; Lafferty & Kuris, 2009; Correia et al., 2023). For all four diversity metrics, we constructed and compared full (interactions between all predictors) and all nested sub-models using AIC, where the full models included host taxa and parasite species as crossed categorical fixed effects. Because the lungworm populations at the two pond sites were homogenous in genetic structure with no significant genetic distance between ponds, and due to a limited amount of sequence data, we pooled data from both sites for these analyses of genetic diversity rather than fitting separate models.

For all four diversity analyses, we included only odonate and frog hosts that had three or more successful parasite sequences per host. For nucleotide diversity, we square-root transformed the remaining estimates and constructed model comparisons as described above using standard GLMs assuming Gaussian errors. For haplotype richness, we compared fits of all models using GLMs with Poisson, negative binomial, and generalized Poisson error distributions to ensure we accounted for any overdispersion in these positive integer data. For haplotype diversity, we fit GLMs with Beta distributions and logit link functions. The Beta distribution is appropriate for continuous data that fall in the interval between 0 and 1, however, we had a few estimates of diversity that were exactly 0 or 1 and thus we rescaled these data using the function ${x}^{{}^{{^{\prime}}}}= \frac{x \left( N-1 \right) +s}{N} $, where *N* is the sample size and *s* is a constant set to 0.5 (see Smithson & Verkuilen, 2006 for details on this approach). Finally, we square-root transformed Shannon–Weiner diversity as well before comparing model fits using standard GLMs with Gaussian errors.

## Results

### Infection prevalence and parasite abundance

We dissected a total of 1,194 *Pseudosuccinea* and *Physa* spp. snails from both research sites and identified at least seven taxa of digenetic trematodes, but only identified *H. complexus* lungworm infections in *Pseudosuccinea*, in spite of previous work indicating that this species can infect both groups of snails (Krull, 1933; Snyder, 1997). It is difficult to discern the reason behind the lack of lungworm detections in the 740 *Physa* spp. we dissected, possibly reflecting some aspect of our study sites that prevented infection in *Physa* spp. *versus Pseudosuccinea*; yet it could also suggest taxonomic issues in past identifications of lungworms in snail hosts, since trematode taxonomy is greatly understudied and detections of species complexes are common (De León & Nadler, 2010; Cribb, 2016). In any case, data from the *Physa* sp. snails (*n* = 740) were excluded for all analyses given no lungworms were detected in these snails. Density of *Planorbella* snails at Lowe Pond was exceedingly low; thus, we only collected a total of 19 individuals throughout the project. Host numbers, parasite infection prevalence, and mean infection abundances for all host stages across both sites are summarized in Table 1.

**Figure 2:**
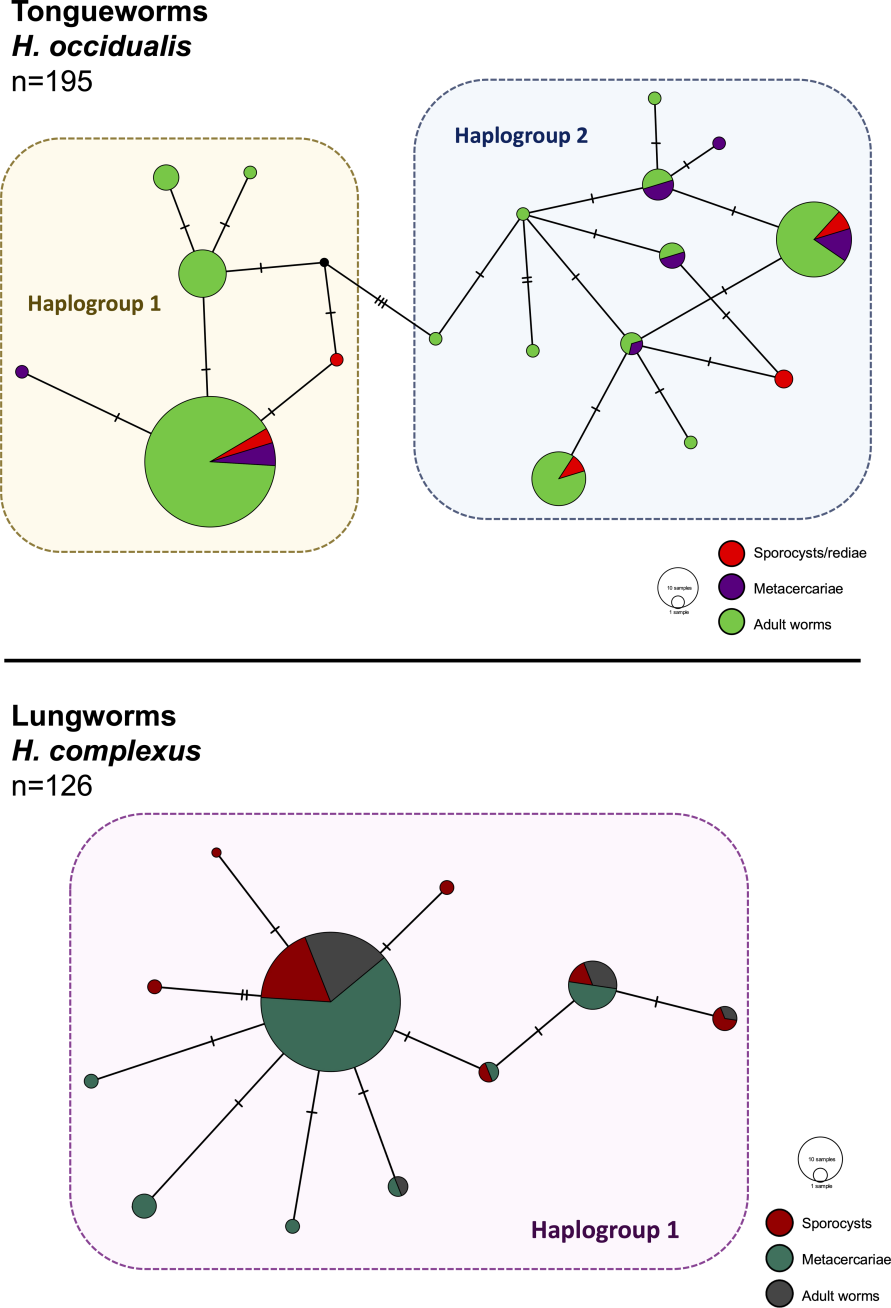
Haplotype networks generated from cytochrome oxidase subunit I (*cox1*) for *H. occidualis* tongueworms (top, 526 bp) and *H. complexus* lungworms (bottom, 519 bp). Colors correspond to life stages. Tongueworms cluster into two distinct haplogroups (number of haplotypes, *n* = 18) while lungworms have lower overall haplotype richness (*n* = 11) forming a single haplogroup.

### Genetic analyses

We identified 18 unique haplotypes comprising two distinct haplogroups in the tongueworm population, and 11 unique haplotypes comprising just one haplogroup in the lungworm population (Fig. 2). Population fixation index *F*_ST_for tongueworms was significant at 0.14352 (*p* < 0.001, Table S1), indicating relatively high levels of genetic differentiation within the population (Merilä & Crnokrak, 2001). Population *F*_ST_ was non-significant and slightly negative for lungworms (*p* = 0.3881, Table S1), indicating that there is no statistical evidence for any genetic differentiation within the population, including between the two pond sites. Pairwise *F*_ST_values between Haplogroups 1 and 2 for tongueworms are also provided in Table S1.

Haplotype rarefaction curves for tongueworms estimated a total of 57.21 haplotypes in the overall population, much higher than the estimated 20.86 haplotypes for lungworms (Table 2; Fig. S1) and consistent with observed haplotype richness. When curves were constructed for metacercariae in odonates and adult worms in frogs separately, for tongueworms estimated haplotype richness was over twice as high in frogs than in odonates (Table 2), but the opposite was true in lungworms; estimated richness was five times higher for metacercariae than adult flukes (Table 2), although this is likely an artifact of uneven sample size.

Host- and population-level haplotype richness and diversity, nucleotide diversity, Tajima’s D, and Fu’s Fs neutrality values for both parasites are also summarized in Table 2. Tajima’s D and Fu’s Fs were non-significant for the tongueworm population (*p* = 0.88 and 0.44, respectively) but significantly negative for lungworms (*p* < 0.001, Table 2), suggesting that the lungworm population had a recent population size expansion potentially following a population reduction due to bottlenecking or a selective sweep (Schmidt & Pool, 2002; Fischer et al., 2006; Yanagida et al., 2012).

### Phylogenetic analysis

**Figure 3:**
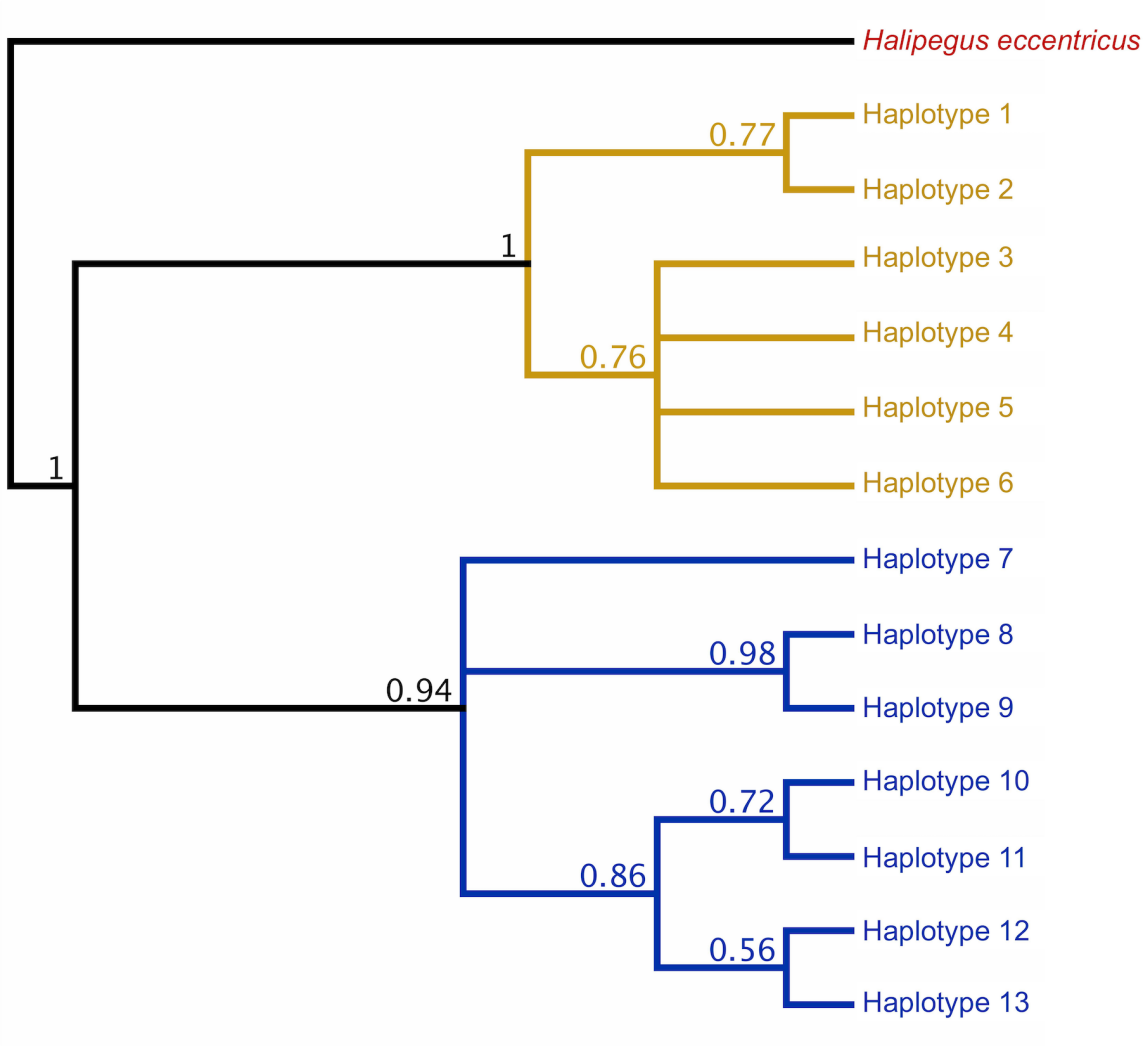
Rooted COI Bayesian phylogenetic tree for a subset of the *Halipegus occidualis* tongueworm population (*n* = 158), with congener *Halipegus eccentricus* as an outgroup. Since this tree was constructed using only sequences with 900+ bp (*n* = 158), we note that the 13 haplotypes shown on this tree are distinct from Haplotypes 1–18 resolved in previous analyses and shown in Fig. 1 that used shorter sequences (*n* = 195, 526 bp). The divergence between the two haplogroups in this population (Haplogroup 1, top, shown in yellow; Haplogroup 2, bottom, shown in blue) was again recovered.

Initial Bayesian trees constructed for both the lungworm and tongueworm populations had low nodal support for most clades across all iterations, resulting in polytomies (Fig. S2). For lungworms, we were only able to resolve relationships between Haplotypes 9, 10, and 11, while for tongueworms we were able to somewhat resolve one haplogroup and one major division between Haplotype 7 and the rest of the resolved haplogroup; thus, we were unable to confidently conclude overall topology for either species but include results from the phylogenetic analysis in Fig. S2. However, when we constructed trees from a subset of the tongueworm population with longer sequence lengths (*n* = 158 sequences of 905 bp, making up 13 new haplotypes), we were able to construct a tree that resolved some clades as moderately- to highly-supported (Fig. 3). Using this extended dataset, we again recovered the divergence between Haplogroups 1 and 2 for this species.

### Trophic accumulation and within-host genetic diversity

**Figure 4:**
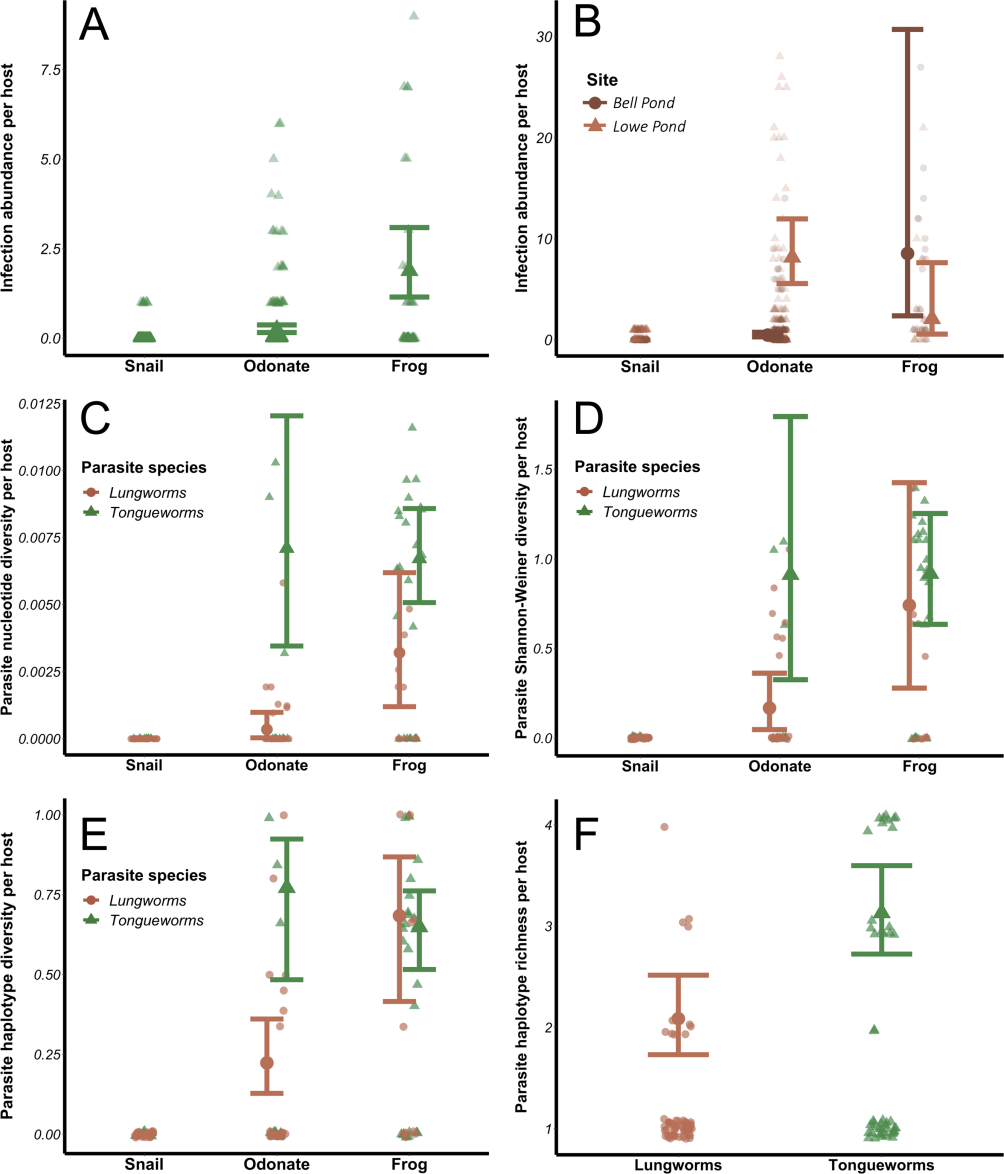
Effects of host trophic level and parasite species on parasite aggregation (A and B) and within-host genetic diversity (C–F). All genetic diversity indices were calculated for individual host assemblages. Error bars represent model results and 95% confidence intervals plotted over raw data points. Data from snails was removed from all four diversity analyses; raw data from snails is plotted for visualization purposes.

Infection abundances for tongueworms increased with host trophic level (Fig. 4A), as did lungworm infection abundances at Bell Pond (Fig. 4B), following the expected pattern of trophic accumulation of parasites in predator hosts (Chen et al., 2008). For lungworms, there were significant differences in parasite population structure between the two pond populations; specifically, at the Lowe site odonates generally carried more lungworm parasites per host than did frogs (Fig. 4B; Table 1). The top four models for analyses of both parasites, dAICc scores, and weights are provided in Tables S2 and S3.

**Figure 5:**
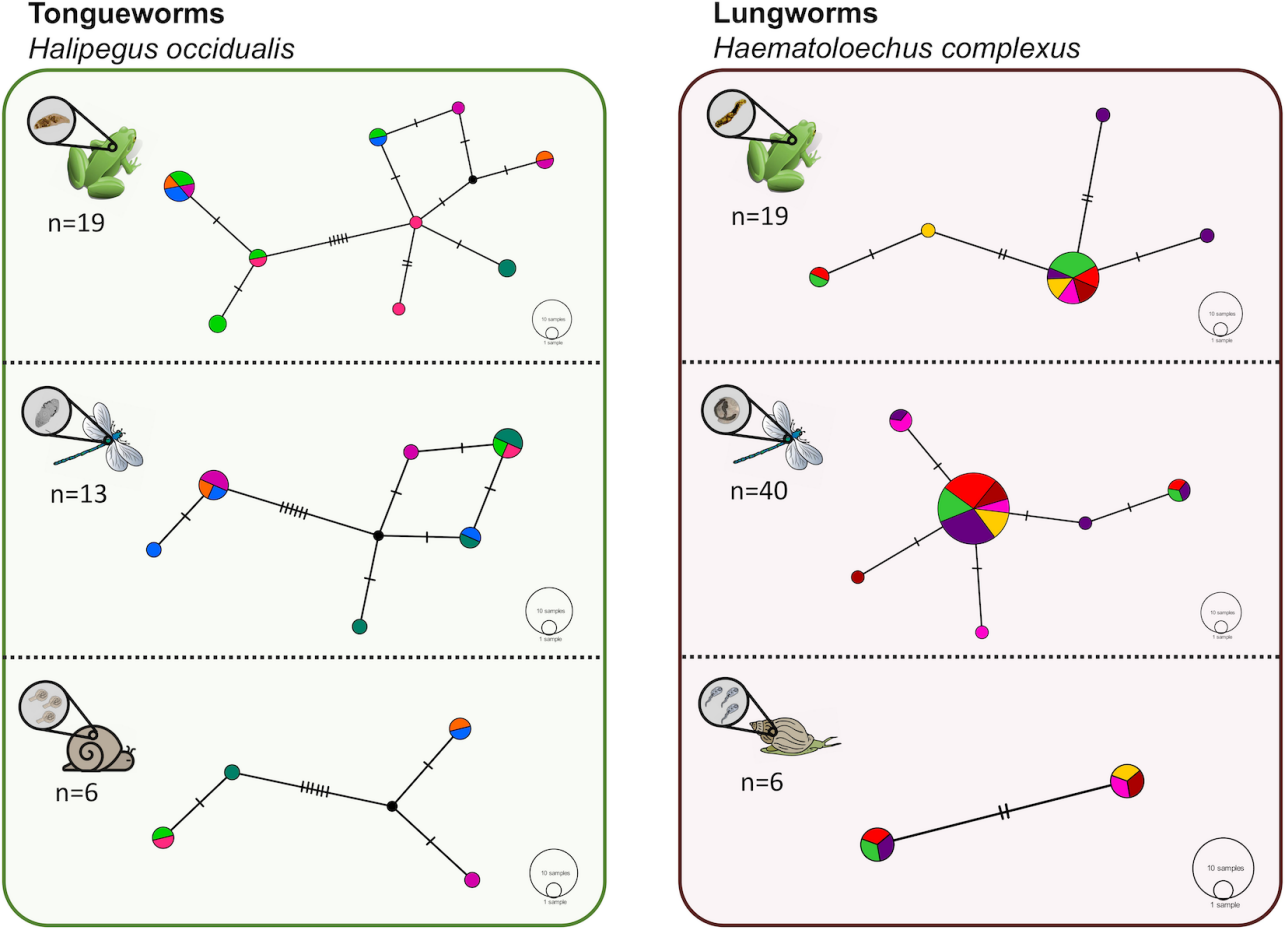
Haplotype networks for subsets of the tongueworm (left) and lungworm (right) parasite populations to visualize haplotype richness by life stage. Six individual hosts (represented by distinct colors) were randomly subsampled from each life stage, per parasite, to partially control for uneven sample sizes across life stages. Overall host-level richness and network complexity generally increased from first-intermediate snail host to definitive frog host, concurrent with parasite accumulation at higher trophic levels. The divergence between haplogroups in the tongueworm population is again apparent here within each host stage.

The relationship between within-host genetic diversity, host stage, and parasite species was dependent on the diversity index. Specifically, host trophic level and parasite species were both predictors of nucleotide diversity *π*. In lungworms, frogs had more diverse parasite assemblages than odonates, which had more diverse assemblages than snails (Fig. 4C)—noting that as previously discussed, snails were not included in the models since they necessarily have a nucleotide diversity of 0. In contrast, for tongueworms there was no detectable difference in estimated nucleotide diversity between odonates and frogs (Fig. 4C). Within-host nucleotide diversity was also approximately seven times higher in odonates and twice as high in frogs for tongueworms as compared to lungworms (Fig. 4C), aligning with expectations from the constructed haplotype networks for both species. Mean Shannon–Weiner diversity of lungworm assemblages increased by almost three times from odonates to frogs, but tongueworm assemblages in frogs had approximately the same mean Shannon–Weiner diversity as those in odonates (Fig. 4D). Tongueworm diversity was also three times higher than lungworm diversity in odonates (Fig. 4D) but not in frogs. For haplotype diversity *h*, diversity tripled with increasing host trophic level to frogs for lungworms, but there was no measurable increase for tongueworms (Fig. 4E). Again, within-host diversities only differed between the two species in odonates, with tongueworm assemblages having three times higher diversity than lungworm assemblages (Fig. 4E). Finally, mean haplotype richness of parasite assemblages only differed between parasite species but not host trophic level (Fig. 4F), where within-host haplotype richness across all host stages was almost 50% higher in tongueworms than in lungworms. The top four models for all four analyses and associated dAICc scores and weights are provided in Table S4 through Table S7. Haplotype networks constructed with a random subset of six hosts per life stage, per parasite species, are presented in Fig. 5 to visualize increasing within-host haplotype diversity at higher host trophic levels.

## Discussion

For parasites with complex life cycles, especially those in isolated spatial patches, maintaining genetic diversity is a critical component of long-term population persistence and evolution (Blasco-Costa, Waters & Poulin, 2012). Using two similar parasites as an empirical comparative study system, we show that trophic transfer of multi-host parasites generally aggregates genetically distinct parasite individuals in higher trophic level predator hosts, although the magnitude of the effect was dependent on parasite species, the diversity metric used, pond population, and sample size. We also found that the isolated population of tongueworms we sampled had surprisingly high genetic diversity at both the population and within-host levels, while the more common lungworm had much lower diversity. From these results, we hypothesize that the dispersal ability of the first-intermediate host for the more common parasite and historic patterns of population isolation for the rarer parasite are potentially driving the patterns of genetic diversity, divergence, and population structure observed in these populations.

### Impacts of host ecology and dispersal on population-level genetic diversity

Frog lungworms (*Haematoloechus complexus*), which use an abundant and broadly distributed first-intermediate snail host that is known to be an efficient colonizing species (Kappes & Haase, 2012; Ebbs, Loker & Brant, 2018; Martin et al., 2020), were much less genetically diverse at both the within-host and the population level than frog tongueworms despite sampling two separate ponds (Figs. 2 and 4; Table 2). Lungworms and their lymnaeid snail hosts may represent a “genetic paradox”, which occurs when a species is efficient at spreading and adapting to new habitats yet has low genetic diversity within colonized patches (Roman & Darling, 2007; Estoup et al., 2016; Schrieber & Lachmuth, 2017; Fowler, Loonam & Blakeslee, 2022). Genetic paradox species also necessarily exhibit founder effects, where the standing genetic variation in a given population is dependent on a limited number of colonists, leading to a bottleneck in the newly colonized population (Barton & Charlesworth, 1984; Blakeslee et al., 2020). Some multi-host parasite species may be particularly susceptible to founder effects due to their dependence on low-trophic level hosts for dispersal (Shoop, 1988; Blakeslee et al., 2020). For example, Blakeslee et al. (2020) found that trematode species in *Ilyanassa* snails showed significantly reduced genetic diversity in introduced populations while their snail hosts did not. Moreover, the dispersal ability of the definitive host and parasite life cycle structure played important roles in these patterns, whereby parasites with simpler life cycles and bird (*i.e.,* highly dispersing) final hosts exhibited weaker founder effects than parasites with more complex life cycles and final hosts with more limited dispersal, *i.e.,* fish (Blakeslee et al., 2020).

Lungworm and tongueworm parasites infect many of the same species of frogs as definitive hosts, which have high site fidelity and short dispersal distances (Durham & Bennett, 1963; Zelmer, Wetzel & Esch, 1999). However, in contrast to their definitive host, the first-intermediate snail hosts of lungworms are highly vagile dispersers that regularly colonize new habitats through multiple pathways (Brown, 1979; Vinarski, 2017; Martin et al., 2020). For example, snails can survive ingestion by waterfowl and colonize new ponds upon excretion, or attach to waterfowl externally (Wesselingh, Cadée & Renema, 1999; Van Leeuwen et al., 2012; Martín-Vélez et al., 2021). Additionally, snails such as *Pseudosuccinea* are able to survive in a wide range of aquatic habitats and are thus expected to be able to establish successful populations after stochastic dispersal events such as waterfowl-driven movement, flooding events, or even anthropogenic sources of dispersal (Van Leeuwen et al., 2013; Lounnas et al., 2017; Vinarski, 2017). Therefore, the lungworm species may be more commonly dispersed *via* movement of snails among sites rather than frogs or even odonate insects, which also often remain at their natal pond for their entire lives (Geenen et al., 2000). Frequent snail dispersal among sites could have a homogenizing effect on the overall lungworm population, reducing genetic structure between pond metapopulations as we observed, and along with potential founder effects from low-diversity colonists may result in the low levels of genetic variation that we found between and within subpopulations (García-Ramos & Kirkpatrick, 1997; Blasco-Costa & Poulin, 2013).

In general, population dynamics of the first-intermediate host snail may play a particularly large role in parasite genetic population structure due to the fact that these hosts produce large numbers of often clonal cercariae (likely hundreds of thousands to millions over a single snail’s lifetime), contributing an outsized amount of parasite propagules to the population over time (Prugnolle et al., 2005; Keeney, Waters & Poulin, 2007a). Although much of their life cycle is similar to lungworms, frog tongueworms require a first-intermediate host that is uncommon, has low dispersal rates, and is a poor colonizer (Martin et al., 2020). The tongueworm’s first-intermediate planorbid snail host is generally characterized as having highly isolated populations with little emigration with other gene pools (Fernandez et al., 2010; Martin et al., 2020). Thus, we expected the tongueworm population to have low genetic diversity and that there would be evidence for genetic bottlenecks due to founder effects and population isolation (Frankel & Soulé, 1981; Epps et al., 2005; Hedrick, 2009). However, overall nucleotide diversity in tongueworms was two times higher and overall haplotype diversity was almost three times higher than that of the lungworm population (Table 2). Accordingly, within-host diversity metrics were also generally higher for both odonate and frog life stages, although this difference more pronounced in odonate hosts depending on the metric used (Table 2, Fig. 4). Interestingly, we saw unexpected genetic divergences within the population of tongueworms, creating two distinct haplogroups separated by significant genetic difference (mean pairwise *F*_ST_ = 0.53, *p* ≤ 0.001) (Figs. 2, 3, and 5; Table S1). The genetic structure we observed in the tongueworm population was unexpected because isolated populations frequently have lower diversity due to founder effects, inbreeding, genetic bottlenecking, and population instability (Frankel & Soulé, 1981; Eckert, Samis & Lougheed, 2008). The tongueworm population sampled in this study is the only population we were able to locate in a large area of eastern North Carolina after rigorous sampling efforts, and the ramshorn snail is also uncommon in this region; both likely due to recent extirpation events and habitat loss (SR Goodnight, pers. obs., 2021; Russell et al., 2015). We cannot rule out the possibility that the patterns of genetic diversity we observed within this tongueworm population may be due to higher gene flow than expected, resulting from introductions of new parasite genetic material through stochastic immigration events from other populations that we did not discover (Lowe & Allendorf, 2010; Fitzpatrick et al., 2016; Kronenberger et al., 2017). However, when originally selecting sites for this study, we surveyed every accessible pond in the nearby area for ramshorn snails and tongueworms in adult frogs, and additionally investigated every iNaturalist (v3.2.7, https://www.inaturalist.org/) record of ramshorn snails in a 200-mile radius for any present tongueworm population (*n* = 13 recorded observations at the time of sampling). The Lowe pond site surveyed for this study was the only tongueworm population we found after visiting numerous sites and all previously documented populations. Therefore, although we cannot entirely rule out immigration of infected hosts from other ponds and the associated gene flow driving these patterns, we expect that such events are at least uncommon at this site. We thus hypothesize that repeated spatial isolation of tongueworm populations in their evolutionary history may have generated historic population divergences, which occur when gene flow from other populations is not sufficient to counteract genetic differentiation caused by genetic drift or selection (Slatkin, 1993; Rousset, 1997; Bolnick & Otto, 2013; Wang & Bradburd, 2014). When we examined a subset of our tongueworm sequences that were longer (905 bp), the resulting phylogenetic tree corroborates the genetic divergence seen in our haplotype network (Figs. 2, 3, and 5). Such deep divergences may be historic, taking place in the evolutionary history of this species, and yet are still apparent even in a small, segregated population presumably colonized by small cohorts of individuals.

We also hypothesize that temporal differences in seasonal ecology of the first-intermediate host snails may have played an additional role in the patterns in genetic diversity that we observed in these two parasite populations. Specifically, lungworms infect lymnaeid pond snails as their first obligate host, which are characterized by “boom and bust” seasonal population dynamics. Specifically, lymnaeids have short life spans (generally a matter of months under natural conditions) and experience mass die-offs and alternating large juvenile recruitment events throughout the summer (Brown, 1979; Esch & Fernandez, 1994; Bargues et al., 2021). In fact, for this study we collected only 1 individual lymnaeid snail during the month of August 2021 since a seasonal die-off in mid-late summer had wiped out most of the resident population. Ponds are thus recolonized by a limited population of estivating snails and their associated parasites the following year, or potentially by dormant eggs persisting through the winter season, leading to repeated bottlenecks of both snail and parasite populations on a yearly or semi-yearly basis (Brown, 1979; Esch & Fernandez, 1994). Even high gene flow between ponds is likely insufficient to overcome recurrent genetic bottlenecks generated by these extreme population fluctuations (Slatkin, 1993; Rousset, 1997; Bolnick & Otto, 2013; Wang & Bradburd, 2014). Consistent with this idea, both Tajima’s D neutrality index and Fu’s Fs were negative for the lungworm population (Table 1), which may indicate relatively rapid population expansion in lungworms, potentially in response to a recent-past genetic bottleneck event or a population crash (Schmidt & Pool, 2002; Fischer et al., 2006; Yanagida et al., 2012). This is in accordance with the expectation that lungworm populations experience highly dynamic seasonal population oscillations driven largely by die-offs of their short-lived, obligate freshwater snail host.

In contrast, Tajima’s D and Fu’s Fs values for the tongueworms were statistically equivocal (Table 1), thus we cannot infer much about the history of the population with these data. However, unlike the large seasonal population fluctuations experienced by the lungworm’s first-intermediate host, tongueworms infect planorbid snails which have 2- to 3-year lifespans and frequently survive through winters to remain at the same pond for multiple consecutive seasons (Goater et al., 1989; Keas & Esch, 1997; Martin et al., 2020). Although planorbid snail populations are also subject to fluctuations resulting from the dynamic seasonal nature of freshwater ponds, tongueworm populations in general likely do not experience the same level of seasonal fluctuation and subsequent bottlenecks experienced by lungworm parasites. The relative population stability of these snails may lead to the retention of rare alleles and signatures of historic genetic divergences within the population over time. The temporal differences in generation time, life history, and population dynamics of the first-intermediate host may play an outsized role in dictating the genetic diversity and structure of patchy freshwater trematode populations.

Finally, one additional possibility contributing to the distinct patterns of genetic structure and diversity we observed between these two parasite species is that adult tongueworms may be more disposed to sexual reproduction than adult lungworms, which may have a higher propensity to self-fertilize (*i.e.,* “selfing”) than to sexually reproduce with a partner. High rates of sexual reproduction in the parasite population would result in higher genetic diversity and potentially genetic divergence between or within populations, as we saw in our study, as compared to high rates of selfing (Criscione & Blouin, 2006; Otto, 2008; Herrmann et al., 2014). Although relative rates of sexual reproduction *vs.* self-fertilization have not been evaluated in our two focal species, certain trematode groups demonstrate variable preferences for alternate reproductive strategies depending on environmental conditions, infection abundances within hosts, and potentially coinfection by related parasites, which dictates much of their genetic population structure (Prugnolle et al., 2005; Criscione & Blouin, 2006; Joannes et al., 2014; Herrmann et al., 2014). For example, Herrmann et al. (2014) demonstrated that some lineages of facultatively progenetic trematodes self-fertilize more often than other lineages, leading to reduced genetic diversity in such lineages. Additionally, we generally found tongueworms “clumped” in the mouth of their definitive host frogs, frequently in groups of 2 or more, while our observations of lungworms had no such clustered distribution; this may suggest that tongueworms have a higher rate of sexual reproduction with partners, potentially contributing to the genetic population structure we observed in this species. Therefore, future research should focus on characterizing innate differences in reproductive strategies between parasite species to more fully understand the mechanisms driving large-scale genetic patterns.

### Impacts of trophic transfer and the complex life cycle on within-host genetic diversity

The evolution of complex life cycles seems counterintuitive; obligate reliance on multiple disparate host species to complete a single life cycle comes with inherent risk that one or more required hosts may be absent in otherwise ideal habitats, preventing population persistence (Parker et al., 2003). This risk may indeed be magnified in parasites that occupy habitats with inherent patchiness, where successful colonization events of new patches as well as immigration between patches are necessary for persistence (Lafferty, 2012; Bitters et al., 2022). However, the aggregation of multiple, genetically distinct parasites into a single predator host through trophic accumulation allows individual hosts to become dispersal mechanisms of genetically diverse cohorts of parasites and enhances the potential for genetic recombination during sexual reproduction (Choisy et al., 2003; Keeney, Waters & Poulin, 2007a), a critical evolutionary advantage of the multi-host life cycle. In accordance with these expectations, we found that tongueworm and lungworm parasite infection abundances (in number of parasites per host individual) were generally higher at higher host trophic levels (*i.e.,* insect *vs.* frog hosts; Figs. 4A and 4B). The population of lungworms at Lowe Pond showed a partially inverted pattern of abundance, however, where individual odonate insects carried approximately four times as many lungworm parasites on average than frogs did adult worms (Table 1; Fig. 4B).

Aggregation of parasites in frog predator hosts was also generally associated with increased genetic diversity at higher host trophic levels (Figs. 4C, 4D and 4E). However, the specific relationship between within-host parasite diversity and host trophic level varied for different diversity indices and differed between parasite species. Specifically, haplotype richness did not differ by host taxa, only by parasite species (Fig. 4F). Haplotype richness is more sensitive to sample size than other metrics, since it is a simple count of unique haplotypes, thus the fact that we had uneven sample sizes between and within hosts across life stages (Table 2) is likely one reason for this discrepancy. In contrast, mean nucleotide diversity, Shannon–Weiner diversity, and haplotype diversity all generally increased with host trophic level for lungworms, even though at one of our pond sites frogs had fewer worms on average than their lower-trophic level insect prey (Figs. 4C, 4D and 4E). This demonstrates the importance of trophic transfer from insects to frogs as critical for maintaining genetic diversity in lungworm populations, where individual frogs consume many infected odonates and therefore accumulate genetically-distinct adult worms for sexual reproduction.

In contrast, odonates hosted equally genetically-diverse tongueworm assemblages as frog hosts, with the only assumed increase in genetic diversity taking place between snails and odonates (Figs. 4C, 4D and 4E). This may highlight the evolutionary advantage of an extra trophic transmission step in the life cycle, in this case between zooplankton and odonates (Fig. 1) (Wetzel & Esch, 1996; Zelmer & Esch, 1998a; Zelmer, Wetzel & Esch, 1999). Zooplankton actively forage, and in doing so may encounter multiple infected snails; thus, a single zooplankton may be infected with multiple tongueworm lineages, though we were unable to assess this by obtaining tongueworm sequences from individual zooplankton. Likewise, odonate predators consume many zooplankton individuals over the course of their larval ontogeny (Benke, 1976; Zelmer & Esch, 1998a). Tongueworms are then afforded an additional opportunity for aggregation in zooplankton, and again in odonate insects, as a result of this additional trophic transfer. In contrast, because lungworms have a very short-lived, free-swimming cercarial stage, we expected infections of single insect hosts to be more spatially limited and thus more likely to consist of cohorts of clonal infections from single snails. The accumulation of clonal cohorts of metacerariae in second intermediate hosts has been documented in other invertebrate species such as amphipods and crabs (Keeney, Waters & Poulin, 2007a; Keeney, Waters & Poulin, 2007b), and although genetic divergence between cohorts has been observed and can be relatively high (Keeney, Waters & Poulin, 2007a; Keeney, Waters & Poulin, 2007b), we did not observe this trend in our data.

Lungworm infection abundances in odonates were much higher than tongueworms (Table 1; Figs. 4A and 4B), with individual odonates frequently infected with 100+ lungworm cysts; the relatively lower genetic diversity of lungworm assemblages in odonates in spite of this abundance presents an additional line of evidence that these parasites may have been predominantly recruited from “clouds” of clonal cercariae produced by single snails. And although there were higher lungworm infection abundances in odonates than in frog hosts (Fig. 4B), the genetic diversity of lungworm assemblages generally did not follow this same trend and instead increased steadily with host trophic level (Figs. 4C, 4D and 4E). This again indicates that the trophic transmission infection pathway is a powerful tool for assembling diverse parasite infrapopulations within predator hosts, thus, we suggest that the two trophic transfers in the tongueworm life cycle act as cumulative mechanisms to maintain genetic diversity in isolated tongueworm populations.

Within-host diversity in large part aligned with overall population diversity metrics, in that tongueworm assemblages were consistently more genetically diverse than lungworm assemblages since the available pool of potential haplotypes was larger for tongueworms than for lungworms (Table 2). Mean nucleotide diversity (Fig. 4C), Shannon–Weiner diversity (Fig. 4D), and haplotype diversity (Fig. 4E) of parasite assemblages in odonates were all generally higher for tongueworms than for lungworms, while there was only a significant difference in frog assemblages for nucleotide diversity (Fig. 4C) where tongueworm assemblages were over twice as diverse on average. For haplotype richness, tongueworm assemblages were more diverse than lungworms but there was no effect of host taxa, likely due in part to uneven sample sizes across life stages (Tables 1 and 2). These results highlight the importance of calculating multiple indices of diversity that consider both genetic distances between parasite individuals as well as the richness of unique haplotypes contained within a single host.

## Conclusions

Parasites are found in essentially every ecosystem on the planet and have broad effects across ecological scales, from host modification to shaping coevolutionary dynamics (Anderson & May, 1982; Bevier & Gorman Gelder, 2018). Multi-host parasites can be particularly impactful across taxa due to their complex life cycles that involve multiple phylogenetically distant hosts spanning across ecosystem boundaries. Maintaining genetic diversity within hosts and across populations is particularly important for parasites that inhabit patchy, highly dynamic habitats like freshwater ponds, where adaptability to those dynamics is paramount for population persistence. In this study, we present some of the first and longest COI sequences for two understudied freshwater parasite species and evaluate the potential impacts of host seasonal-temporal dynamics, life history, and population isolation on genetic diversity and divergence across scales. We found that higher trophic level hosts were generally infected by more genetically diverse parasite assemblages, although the effect differed based on parasite life history strategy, showcasing a patent benefit of the trophic transmission mechanisms characteristic of multi-host life cycles. We found that an isolated population of *Halipegus occidualis* tongueworms had surprisingly high genetic diversity and structure, and we characterized a historic divergence between two distinct haplogroups in the population. In contrast, *Haematoloechus complexus* lungworm parasites were relatively genetically homogenous with little genetic structure even across two separate pond subpopulations. We suggest that the temporal ecology and life history of the first-intermediate host contribute largely to these patterns.

## Supplemental Information

10.7717/peerj.19178/supp-1 Supplemental Information 1 Tongueworm COI alignment (526bp) Alignment of cytochrome oxidase subunit 1 (COI) mitochondrial gene, 526bp.

10.7717/peerj.19178/supp-2 Supplemental Information 2 Alignment file of lungworm COI sequences (526 bp) Alignment of cytochrome oxidase subunit 1 (COI) mitochondrial gene, 519bp.

10.7717/peerj.19178/supp-3 Supplemental Information 3 Haplotype rarefaction curves for overall tongueworm and lungworm populations (A), tongueworms only (B) and lungworms only (C) Curves were constructed using EstimateS (Version 9, R. K. Colwell, http://purl.oclc.org/estimates; Colwell *et al.* 1997) and plotted using ggplot2 in R (Wickham, 2016).

10.7717/peerj.19178/supp-4 Supplemental Information 4 Bayesian phylogenetic trees of *Halipegus occidualis* (*n* = 195, 526 bp; top panel) and *Haematoloechus complexus* (*n* = 126, 519 bp; bottom panel) parasite populations using COI mitochondrial sequences Trees were constructed using Mr. Bayes (Huelsenbeck and Ronquist 2001) implemented in Geneious version 2023.0.1 (Kearse et al., 2012).

10.7717/peerj.19178/supp-5 Supplemental Information 5 Supplemental statistical tables For all analyses, sample size corrected Akaike Information Criterion (AICc) model comparisons were used to determine best fit. Distribution codes are as follows: LNB-ZI, zero-inflated linear negative binomial; QNB-ZI, zero-inflated quadratic negative binomial; LNB, linear negative binomial; QNB, quadratic negative binomial; N, normal (Gaussian); B, Beta; and G-P, generalized Poisson (log link).

## Additional Information and Declarations

### Field Study Permissions

The following information was supplied relating to field study approvals (i.e., approving body and any reference numbers): Specimens were collected under North Carolina Wildlife Collection Permit #22-SC01489.

### DNA Deposition

The following information was supplied regarding the deposition of DNA sequences: The DNA/sequences are available at GenBank and Figshare: - PQ675873–PQ675998 (*Haematoloechus*) and PQ668820–PQ669020 (*Halipegus sequences*). - DNA alignments: Goodnight, Sarah (2025). FASTA DNA alignment files for “Genetic diversity and population structure of parasite infrapopulations within and across hosts for two trophically transmitted trematode parasites” submitted to PeerJ October 2024. figshare. Dataset. https://doi.org/10.6084/m9.figshare.27304869.v1.

### Data Availability

The following information was supplied regarding data availability: The data is available at Figshare: - Data: Goodnight, Sarah; McCoy, Michael; Blakeslee, April M. H. (2025). Data (.csv files) for ”Genetic diversity and population structure of parasite infrapopulations within and across hosts for two trophically transmitted trematode parasites” submitted to PeerJ October 2024. figshare. Dataset. https://doi.org/10.6084/m9.figshare.23576454.v1
- Statistical code: Goodnight, Sarah (2025). Statistical code (.rmd files) for ”Genetic diversity and population structure of parasite infrapopulations within and across hosts for two trophically transmitted trematode parasites” submitted to PeerJ October 2024. figshare. Dataset. https://doi.org/10.6084/m9.figshare.27304536.v1
- DNA alignments: Goodnight, Sarah (2025). FASTA DNA alignment files for ”Genetic diversity and population structure of parasite infrapopulations within and across hosts for two trophically transmitted trematode parasites” submitted to PeerJ October 2024. figshare. Dataset. https://doi.org/10.6084/m9.figshare.27304869.v1.
