# Supplementary figures and images for: Genetic diversity and population structure of parasite infrapopulations within and across hosts for two trophically transmitted trematode parasites
Source: PeerJ. 2025 Apr 28;13:e19178. doi: 10.7717/peerj.19178 (PMC12045269; doi:10.7717/peerj.19178)

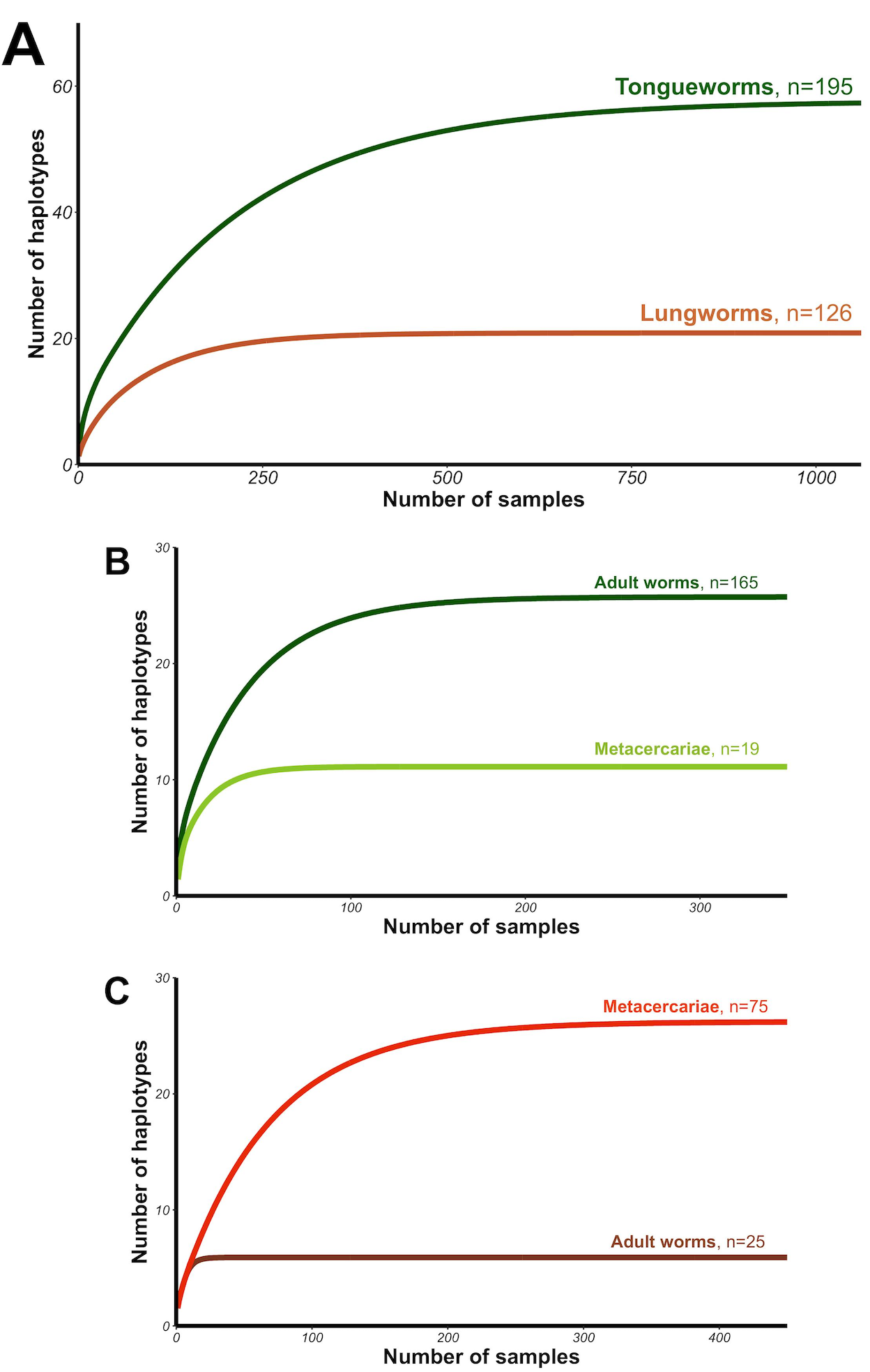

Supplement: Supplemental Information 3 — Curves were constructed using EstimateS (Version 9, R. K. Colwell, http://purl.oclc.org/estimates; Colwell et al. 1997) and plotted using ggplot2 in R (Wickham, 2016). [file peerj-13-19178-s003.png]

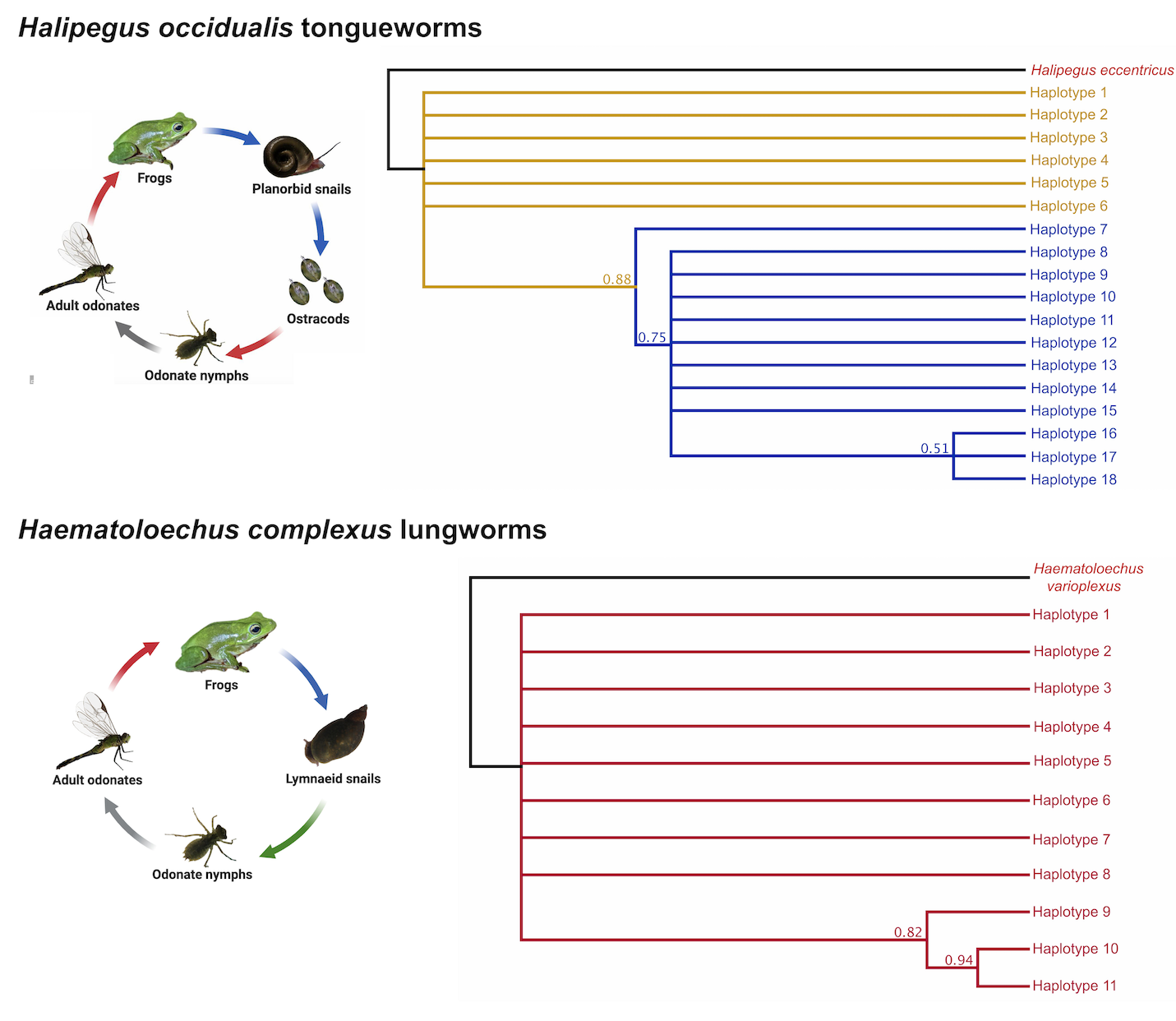

Supplement: Supplemental Information 4 — Trees were constructed using Mr. Bayes (Huelsenbeck and Ronquist 2001) implemented in Geneious version 2023.0.1 (Kearse et al., 2012). [file peerj-13-19178-s004.png]
