# Supplementary material for: Genetic diversity and population structure of parasite infrapopulations within and across hosts for two trophically transmitted trematode parasites
Source: PeerJ. 2025 Apr 28;13:e19178. doi: 10.7717/peerj.19178 (PMC12045269; doi:10.7717/peerj.19178)
Supplement: Supplemental Information 5 — For all analyses, sample size corrected Akaike Information Criterion (AICc) model comparisons were used to determine best fit. Distribution codes are as follows: LNB-ZI, zero-inflated linear negative binomial; QNB-ZI, zero-inflated quadratic negative binomial; LNB, linear negative binomial; QNB, quadratic negative binomial; N, normal (Gaussian); B, Beta; and G-P, generalized Poisson (log link). [file peerj-13-19178-s005.docx]

**Supplementary statistical tables**

For all analyses, sample size corrected Akaike Information Criterion (AICc) model comparisons were used to determine best fit. Distribution codes are as follows: LNB-ZI = zero-inflated linear negative binomial, QNB-ZI = zero-inflated quadratic negative binomial, LNB = linear negative binomial, QNB = quadratic negative binomial, N= normal (Gaussian), B = Beta, and G-P = generalized Poisson (log link).

**Table S2. Top models, dAICc values, and weights for effects of host taxa on tongueworm infection abundance per host.**

| **Model** | **Distribution** | **Rank** | **dAICc** | **Df** | **Weight** |
| --- | --- | --- | --- | --- | --- |
| Infection abundance ~ Host taxa  Infection abundance ~ Host taxa  Infection abundance ~ Host taxa  Infection abundance ~ 1 | LNB  LNB-ZI  QNB-ZI  LNB | 1  2  3  4 | 0.0  1.8  13.4  30.0 | 3  4  4  2 | 0.710  0.290  <0.001  <0.001 |

**Table S3. Top models, dAICc values, and weights for effects of host taxa on lungworm infection abundance per host.**

| **Model** | **Distribution** | **Rank** | **dAICc** | **Df** | **Weight** |
| --- | --- | --- | --- | --- | --- |
| Infection abundance ~ Host taxa * Site  Infection abundance ~ Host taxa * Site  Infection abundance ~ Host taxa + Site  Infection abundance ~ Host taxa + Site | QNB  QNB-ZI  QNB  QNB-ZI | 1  2  3  4 | 0.0  2.0  10.8  12.8 | 5  6  4  5 | 0.7277  0.2677  0.0033  0.0012 |

**Table S4. Top models, dAICc values, and weights for impacts of host taxa and parasite species on within-host nucleotide diversity π.**

| **Model** | **Distribution** | **Rank** | **dAICc** | **Df** | **Weight** |
| --- | --- | --- | --- | --- | --- |
| log(π) ~ Host taxa * Parasite species  log(π) ~ Host taxa + Parasite species  log(π) ~ Parasite species  log(π) ~ Host taxa | N  N  N  N | 1  2  3  4 | 0.0  3.0  5.2  17.7 | 5  4  3  3 | 0.770  0.171  0.059  <0.001 |

**Table S5 Top models, dAICc values, and weights for impacts of host taxa and parasite species on within-host Shannon-Weiner diversity H’.**

| **Model** | **Distribution** | **Rank** | **dAICc** | **Df** | **Weight** |
| --- | --- | --- | --- | --- | --- |
| sqrt(H’) ~ Host taxa * Parasite species  sqrt(H’) ~ Host taxa + Parasite species  sqrt(H’) ~ Parasite species  sqrt(H’) ~ Host taxa | N  N  N  N | 1  2  3  4 | 0.0  0.8  1.9  3.1 | 5  4  3  3 | 0.442  0.295  0.169  0.094 |

**Table S6. Top models, dAICc values, and weights for impacts of host taxa and parasite species on within-host haplotype diversity *h*.**

| **Model** | **Distribution** | **Rank** | **dAICc** | **Df** | **Weight** |
| --- | --- | --- | --- | --- | --- |
| *h* ~ Host taxa * Parasite species  *h* ~ Parasite species  *h* ~ Host taxa + Parasite species  *h* ~ Host taxa | B  B  B  B | 1  2  3  4 | 0.0  4.7  4.9  6.0 | 5  3  4  3 | 0.8103  0.0768  0.0696  0.0412 |

**Table S7. Top models, dAICc values, and weights for impacts of host taxa and parasite species on within-host haplotype richness *h_rich_*.**

| **Model** | **Distribution** | **Rank** | **dAICc** | **Df** | **Weight** |
| --- | --- | --- | --- | --- | --- |
| *h_rich_* ~ Parasite species  *h_rich_* ~ Host taxa + Parasite species  *h_rich_* ~ Host taxa * Parasite species  *h_rich_* ~ Host taxa | G-P  G-P  G-P  G-P | 1  2  3  4 | 0.0  1.4  2.5  3.4 | 3  4  5  3 | 0.5083  0.2529  0.1441  0.0914 |
